# Genotyping-by-sequencing and genomic selection applications in hexaploid triticale

**DOI:** 10.1093/g3journal/jkab413

**Published:** 2021-12-13

**Authors:** Habtamu Ayalew, Joshua D Anderson, Nick Krom, Yuhong Tang, Twain J Butler, Nidhi Rawat, Vijay Tiwari, Xue-Feng Ma

**Affiliations:** 1 Noble Research Institute, LLC., Ardmore, OK 73401, USA; 2 Department of Agronomy, Kansas State University, Manhattan, KS 66506, USA; 3 Department of Plant Science and Landscape Architecture, University of Maryland, College Park, MD 20742, USA; 4 Forage Genetics International, West Salem, WI 54669, USA

**Keywords:** genotyping-by-sequencing (GBS), genomic selection (GS), linkage disequilibrium (LD), population genetics, triticale

## Abstract

Triticale, a hybrid species between wheat and rye, is one of the newest additions to the plant kingdom with a very short history of improvement. It has very limited genomic resources because of its large and complex genome. Objectives of this study were to generate dense marker data, understand genetic diversity, population structure, linkage disequilibrium (LD), and estimate accuracies of commonly used genomic selection (GS) models on forage yield of triticale. Genotyping-by-sequencing (GBS), using *Pst*I and *Msp*I restriction enzymes for reducing genome complexity, was performed on a triticale diversity panel (*n *=* *289). After filtering for biallelic loci with more than 70% genome coverage, and minor allele frequency (MAF) >* *0.05, de novo variant calling identified 16,378 single nucleotide polymorphism (SNP) markers. Sequences of these variants were mapped to wheat and rye reference genomes to infer their homologous groups and chromosome positions. About 45% (7430), and 58% (9500) of the de novo identified SNPs were mapped to the wheat and rye reference genomes, respectively. Interestingly, 28.9% (2151) of the 7430 SNPs were mapped to the D genome of hexaploid wheat, indicating substantial substitution of the R genome with D genome in cultivated triticale. About 27% of marker pairs were in significant LD with an average *r*^2^* *>* *0.18 (*P < *0.05). Genome-wide LD declined rapidly to *r*^2^ < 0.1 beyond 10 kb physical distance. The three sub-genomes (A, B, and R) showed comparable LD decay patterns. Genetic diversity and population structure analyses identified five distinct clusters. Genotype grouping did not follow prior winter *vs* spring-type classification. However, one of the clusters was largely dominated by winter triticale. GS accuracies were estimated for forage yield using three commonly used models with different training population sizes and marker densities. GS accuracy increased with increasing training population size while gain in accuracy tended to plateau with marker densities of 2000 SNPs or more. Average GS accuracy was about 0.52, indicating the potential of using GS in triticale forage yield improvement.

## Introduction

Triticale (*× Triticosecale* Wittmack) is a man-made cereal species developed through the hybridization of wheat (*Triticum* spp.) and rye (*Secale cereale* L.) for combining the hardiness and nutrient-use efficiency of rye and grain yield and quality of wheat. It can have different ploidy levels and genome compositions ranging from tetraploid to octoploid depending on the type of wheat parent involved in the hybridization (Ayalew *et al.* 2018). Hexaploid triticale (2*n* = 42 = AABBRR) is the most commonly cultivated form because of its better genomic stability and superior agronomic performance (Ammar *et al.* 2004; Oettler 2005). Triticale has a large and complex genome (∼17 Gb) with some degree of outcrossing (Oettler 2005).

Triticale is mainly grown for animal feed and cover crop (Newell and Butler 2013; Ayalew *et al.* 2018). As a result, current triticale breeding efforts in the southern Great Plains of the United States are mainly focused on biomass yield and biotic/abiotic stress resistance improvement (Saha *et al.* 2015; Kim *et al.* 2017; Kumssa *et al.* 2019). Triticale breeding can benefit from the contemporary developments in molecular markers and genomics-assisted breeding, but marker development for genetics and breeding studies in triticale has lagged behind other cereal crops. Most of the markers used in triticale were derived from either wheat or rye while only limited number of markers were directly developed from triticale (Kuleung *et al.* 2004; Badea *et al.* 2011).

The advent of high-throughput sequencing technologies enabled relatively easy and rapid marker development, even in highly complex and large genomes (Craig *et al.* 2008; Huang *et al.* 2009; Elshire *et al.* 2011; Poland *et al.* 2012b; He *et al.* 2014; Scheben *et al.* 2017). Genotyping-by-sequencing (GBS), one of the high-throughput genotyping technologies, utilizes restriction enzymes to reduce genome complexity (Elshire *et al.* 2011; Poland *et al.* 2012b; He *et al.* 2014). GBS has been reported to be a highly efficient marker discovery tool in wheat and barley (Poland *et al.* 2012b), rice and soybean (Deschamps *et al.* 2010), maize (Liu *et al.* 2015; Wang *et al.* 2020), oat (Carlson *et al.* 2019), and potato (Bastien *et al.* 2018), thereby facilitating genome-wide association studies (GWAS) and genomic selection (GS) applications.

Developments in statistical genetics enabled utilization of the rapidly increasing marker data in plant breeding and genetics. GS is one of the most promising tools to exploit marker technologies in plant breeding through estimation of breeding values of individuals even before phenotyping (Meuwissen *et al.* 2001; Endelman 2011; Xu *et al.* 2020). Unlike conventional marker-assisted selection (MAS), which uses markers linked to traits of interest as a diagnostic tool, GS computes genomic estimated breeding values (GEBVs) of individuals using all available genome-wide markers regardless of their effects on the phenotype (Goddard 2009; Xu *et al.* 2020). This makes GS an attractive strategy for genetic improvement of highly quantitative and complex traits controlled by many genes with minor effects (Crossa *et al.* 2010; Jannink *et al.* 2010).

GS accuracy varies depending on the underlying population structure, linkage disequilibrium (LD), training population size, and marker density (Meuwissen *et al.* 2001; VanRaden 2008; Endelman 2011). It is a common consensus that increasing training population size increases GS accuracy but the trend of increase declines once the genome is well covered by a set of representative markers (Xu *et al.* 2020; Maulana *et al.* 2021). The extent of LD in a population and its decay distance determines the number of markers to be used (Flint-Garcia *et al.* 2003; Liu *et al.* 2015; Vos *et al.* 2017). Generally, cross-pollinated crops have shorter LD decay distance, as a result, they require larger number of markers compared with self-pollinated crops (Liu *et al.* 2015; Hao *et al.* 2019). Consequently, no single model is universally recommended, making model selection and optimization a necessary step to practice GS in plant breeding.

The objectives of this study were to (1) generate a set of triticale markers using a diversity panel consisting of diverse breeding parents and representative triticale accessions from the National Small Grains Collection (NSGC); (2) characterize the marker set through genome mapping and LD analysis; (3) characterize diversity and population structure of the panel; and (4) evaluate the feasibility of GS in forage triticale breeding.

## Materials and methods

### Plant materials and phenotyping

The experiment started in 2017–2018 season by characterizing 1400 accessions in a paired-row single observation plot of 1.5-m long and 40-cm wide at Gene Autry, Oklahoma, USA. A total of 289 diverse hexaploid triticale lines were selected out of the initial 1400 accessions on the basis of phenology, plant architecture, winter hardiness, biomass, and grain yield representing available diversity to compose an association mapping population. This population consisted of 39 cultivars or elite breeding lines developed primarily from the southern Great Plains, and 250 accessions from the NSGC that are being used as newly introduced parents in our breeding program. Altogether, there are 75 spring, and 196 winter-type lines in the panel. The panel was further evaluated in replicated trials at one location (Burneyville, Oklahoma, USA) in 2018–2019, and two locations (Gene Autry and Burneyville, Oklahoma, USA) in 2019–2020. Triple lattice design with plot size of 2.25 m^2^ was used in the 2018–2019 season while simple lattice with the same plot size was used in the 2019–2020 season. Forage biomass sample was manually harvested from two adjacent rows and total dry weight was converted to g m^−2^ scale.

### DNA isolation, library construction, and sequencing

Genomic DNA of each accession was isolated from bulked fresh leaf samples of 10 days old seedlings immediately frozen in liquid nitrogen. DNA was extracted using DNeasy Plant Mini Kit (Qiagen Inc., USA). The DNA concentration and quality of each sample were assessed before library construction using a nano-photometer (Thermo Fisher Scientific). Genome complexity reduction and multiplexed GBS library construction were performed based on the *Pst*I*–Msp*I method (Poland *et al.* 2012b) with 48× multiplex sequencing libraries in seven plates. DNA sequencing was performed on an Illumina NextSeq 500 platform following standard protocols for paired-end reads.

### De novo read assembly and variant calling

Raw sequence data in FASTQ format were processed using ipyrad_v.0.9.45 software (Eaton and Overcast 2020) with the default assembly parameters to demultiplex, trim, and align the reads, followed by identifying variant calls for generating a VCF file for each plate. A single merged VCF file of all seven plates was then produced by aligning sequences and single nucleotide polymorphism (SNP) calls across all seven plates. The final merged file was further filtered to include only those SNP positions with at least 70% in coverage and 0.05 for minor allele frequency (MAF). Accessions with missing values greater than 30% were removed before downstream analysis. Sequences of the filtered SNPs were mapped to the wheat and rye genomes to identify their genome origins and homoeologous groups (IWGSC 2018; Rabanus-Wallace *et al.* 2021). Of the two recent rye genome references (Li *et al.* 2021; Rabanus-Wallace *et al.* 2021), we used the Lo7 reference because our initial analysis was based on sequences published previously (Bauer *et al.* 2017).

### Linkage disequilibrium analysis

The LD pattern was estimated by using squared allele frequency (*r*^2^) based on loci that have been mapped on the wheat and rye reference genomes (Bauer *et al.* 2017; IWGSC 2018; Rabanus-Wallace *et al.* 2021). Pairwise LD was computed using a sliding window of 50 kb using TASSEL v5.2.50 (Bradbury *et al.* 2007). Genome-wide LD (*r*^2^) was plotted against the physical distance (bp) between markers to determine the LD decay distance (bp). Differences in LD patterns and decay distances were evaluated and compared among the three sub-genomes (A, B, and R). It was assumed that *r*^2^ values higher than 0.1 are likely to be caused by genetic linkage. Mean *r*^2^ values were calculated for every 500 bp interval window. The interval at which the *r*^2^ values fall below 0.1 was taken as LD decay distance. This LD decay distance was supported by fitting a nonlinear smoothing curve (Hill and Weir 1988) and determining the intersection between the smoothing curve and the horizontal line that passes through *r*^2^ = 0.1 (*r*^2^ cut-off value). The expected value of *r*^2^ under drift-recombination equilibrium with a low level of mutation and an adjustment for sample size *n*, was calculated using Hill-Weir’s equation as follows (Hill and Weir 1988),

$${E(r}^{2})=\left[\frac{10+C}{\left(2+C)(11+C\right)}\right]\left[1+\frac{\left(3+C\right)\left(12+12C+C^{2}\right)}{n\left(2+C\right)\left(11+C\right)}\right],$$

where *n* is the effective population size, and *C* is the product of population level recombination (*ρ*) and distance, which can be calculated as *C* = ρ*distance.

### Genetic diversity and population structure analysis

Three population clustering methodologies were used *i.e.*, hierarchical clustering, k-means clustering, and principal components analysis (PCA). Principal components (PCs) were further analyzed by using discriminant analysis of the principal components (DAPCs).

PC analysis was performed using the *dudi.pca* function of the adegenet package in R (Jombart and Ahmed 2011). Missing values were imputed using genome-wide mean. Hierarchical clustering was performed using *hclust* function in R (Murtagh and Legendre 2014). K-means clustering using *find.clusters* function in the adegenet/R was used to identify the number of groups of lines (Jombart and Ahmed 2011). The optimal number of k-means was determined by using the Bayesian information criterion (BIC) as a statistical measure of goodness of fit. Population structure was further described using DAPC with the optimum number of PCs interactively determined using the adegenet/R package with 10^5^ iterations (Jombart *et al.* 2010; Jombart and Ahmed 2011).

### Forage yield data analysis

Data were analyzed in two steps. In the first step, data from each year and location were analyzed separately using agricolae/R package (de Mendiburu 2019) for lattice design analysis to account for incomplete block effects. The same data set was analyzed using a two-dimensional P-spline mixed model to correct for spatial effects using SpATS/R package (Rodríguez-Álvarez *et al.* 2018) and residuals were compared to determine the optimum model for final analysis. Spatial analysis was performed for each environment separately using the following model (Velazco *et al.* 2017): *Y* = *Xβ* + *X_s_β_s_* + *Z_s_S* + *Z_u_U* + *Z_g_G* + *e*, where the vector *Y* contains forage yield arrayed as rows within columns, *β* is a resolvable block effect, and *X* is the associated design matrix. The fixed (unpenalized) term *X_s_β_s_* and the random (penalized) component *Z_s_S* form the mixed model expression of the smooth spatial surface, *i.e.*, *f(r, c)*  = *X_s_β_s_* + *Z_s_S*, where the vector of random spatial effects *s* has covariance matrix *S*. The vector *U* comprises the mutually independent sub-vectors of random row and column effects accounting for discontinuous field variation, with design matrix *Z_u_* =  [*Z_r_*|*Z_c_*] and covariance matrix *U* = diag (*σ^2^_r_I_r_*, *σ^2^_c_I_c_*). The vector *G* contains the random genotypic effects and *Z_g_* is the associated design matrix. We assumed independent genotypic variance, *i.e.*, *g* ∼ *N*(0, *G*), with *G* = *σ^2^_g_I_g_*. The vector *e* consists of spatially independent residuals with distribution *e* ∼ *N*(0, *σ^2^_e_I*). Since spatial analysis significantly reduced residual error, final analysis to obtain predicted means for each environment was done using spatial analysis.

In the second step, combined analysis was performed on adjusted means from each season and location using the following mixed linear model: *Y_ijk_* *=* μ + *G_i_* + *E_j_* + *GE_ij_* + *e_ijk_*, where *Y_ijk_* is the observed mean, *μ* is the grand mean, *G_i_* is the effect of the *i*th genotype, *E_j_* is the effect of the *j*th environment (locations and seasons were considered as separate environments), *GE_ij_* is the effect of the *i*th genotype in the *j*th environment, *e_ijk_* is the random error. Genotypes were considered as fixed effects while environments were considered random.

### Genomic selection model evaluation

GS was performed using the ridge regression best linear unbiased prediction (rrBLUP) R package (Endelman 2011). Three different GS models, RRBLUP using *mixed.solve* function with marker design matrix, GBLUP using *kinship*. *BLUP* with additive relationship matrix, and GUASS model using Euclidean distance matrix, were tested with 10^3^ iterations each. GS accuracy was evaluated as the average correlation value between GEBV and phenotypic estimated breeding value (PEBV).

## Results

### Variant calling and quality control

Average read depth for each individual over all variants was 13, while some individuals showed very low coverage (Supplementary Figure S1A). A total of 204,106 SNP variants were identified via de novo variant calling. Missing value percentage was close to 86% before some low coverage SNPs were filtered out (Supplementary Figure S1B). After filtering for biallelic SNPs with at least 70% genome coverage, and MAF* *>* *0.05, a total of 16,378 biallelic SNPs were identified (Supplementary Figure S1C). Homologous groupings of markers were identified by mapping sequence reads to the wheat (IWGSC 2018), and rye (Rabanus-Wallace *et al.* 2021) reference genomes. Out of the 16,378 SNPs, 30% (4913) were mapped to both wheat and rye reference genomes while the other 15% (2516) were mapped specifically to wheat genome making the total number of SNPs mapped to wheat genome 7430 (45%). Similarly, about 58% (9500) of the 16,378 SNPs were mapped to the rye genome with 28% (4519) being specific to rye. Twenty-eight percent (4510) of the 16,378 SNPs remained unmapped, but were used to analyze genetic diversity as unmapped groups.

Chromosomal locations of SNPs were determined based on the locations of the SNPs mapped to the wheat genome. All the SNPs mapped to the D genome of wheat are hereafter considered as R genome SNPs as triticale does not have a D genome. Out of the 7430 SNPs mapped to wheat reference genome, 31% (2284), 40% (2995), and 28.9% (2151) SNPs were located at A, B, and R sub-genomes, respectively. Similarly, out of the 9500 high-quality SNPs mapped to the rye reference genome, about 6670 SNPs were potentially located on the R sub-genome of triticale (2151 from those mapped to the D genome of wheat and 4519 from those specifically mapped to rye reference genome).

### Linkage disequilibrium

**Figure 1:**
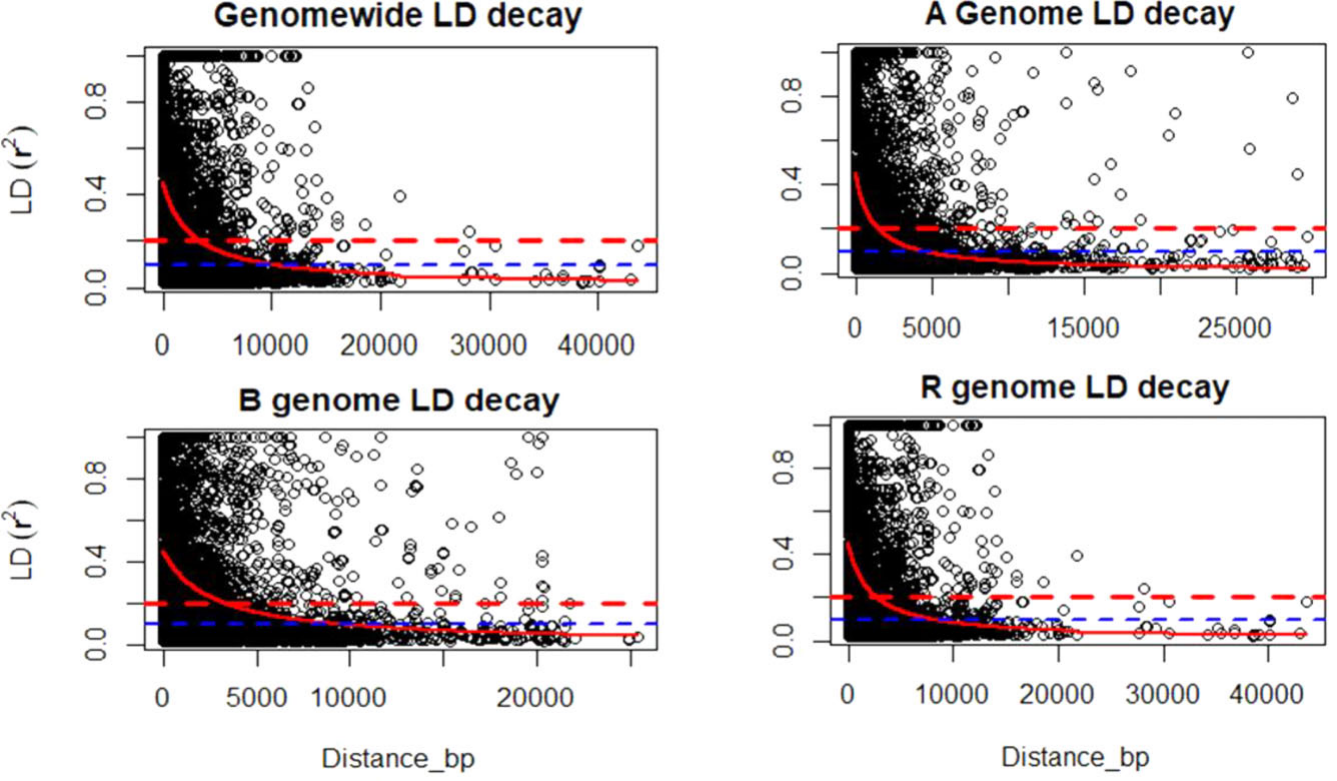
Genome-wide linkage disequilibrium (LD) decay plot in hexaploid triticale. LD, measured as *r*^2^ between pairs of polymorphic sites, is plotted against physical distance (bp) between the sites. LD decayed to *r*^2^ < 0.1 beyond 10-kb distance.

The nature of LD and the extent of its decay distance were investigated using markers that have known location in a genome. Close to 27% of all marker pairs were in significant LD with *r*^2^* *>* *0.18 (*P < *0.05). The *r*^2^ values from the whole genome LD were separated into the respective genomes to compare LD patterns. LD based on the whole genome dropped quickly to *r*^2^* *<* *0.1 at about 10 kb (Figure  1A). About 24% of marker pairs on the A genome were in significant LD (*P < *0.05), while the B and R genomes had 25% and 27% of marker pairs in significant LD. The A genome had the shortest LD block (4 kb) while B and R genomes had similar decay distances in the range of 10 kb (Figure  1, B–D).

### Genetic diversity and population structure

**Figure 2:**
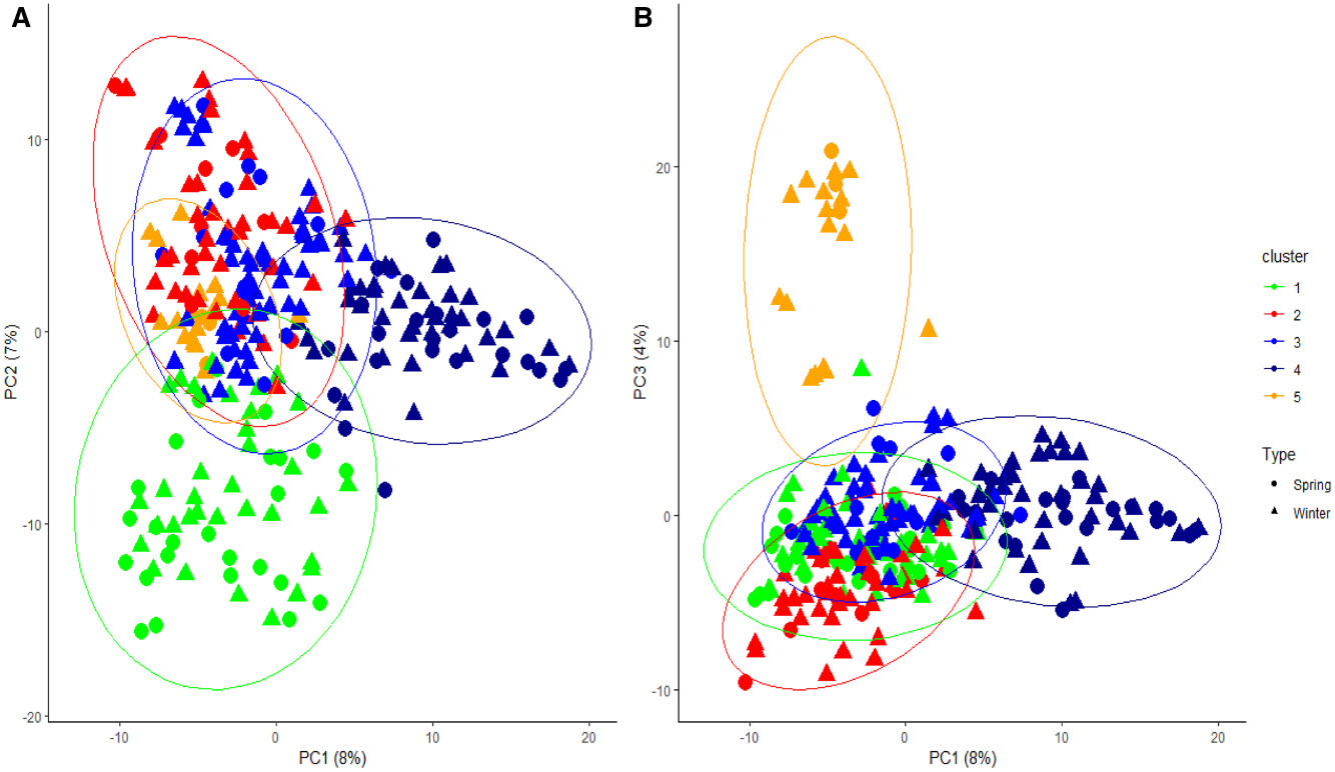
The first three principal components, (A) for PC1 *vs* PC2 and (B) for PC1 *vs* PC3, explained 19% variation of the population. Cluster numbers were based on the k-means output. Each cluster was admixture of both winter and spring types.

**Figure 3:**
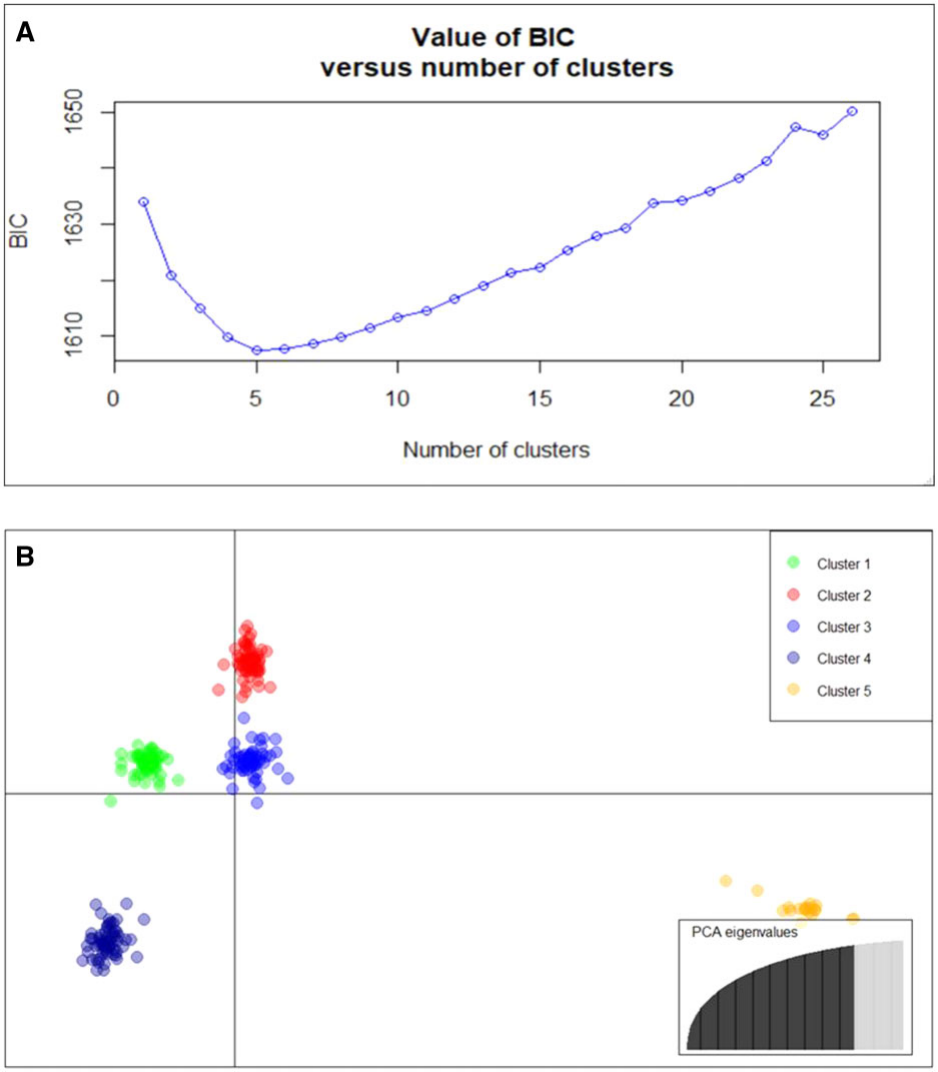
Discriminant analysis of principal components of the triticale panel. (A) The optimal number of k-means was determined using BIC relative to the numbers of clusters (k) tested. (B) Scatter plot of DAPCs showed well-separated clusters through maximizing variation among groups and minimizing variation within groups. The main figure shows the relative scatter of the five clusters, in which each dot represents a unique genotype. The PCA eigenvalue inset (bottom right) indicated that about 95% of variation was captured by using 200 PCs.

Three different methods were used to infer population structure and allelic diversity of the panel. Hierarchical clustering, PCA and DAPC all showed five clusters. Genotypes were grouped into five clusters based on Euclidian distance matrix and Wad.D^2^ agglomeration (Supplementary Figure S2). The first three PCs explained 19% of genetic variation (Figure  2). For *k*-means clustering, a statistical measure of goodness of fit was computed for each *k*-value to assist determining the optimal cluster number. The BIC was the lowest at *k *=* *5 (Figure  3A), indicating five distinct clusters are most suitable for classifying genotypes in the panel. The results of hierarchical clustering and *k*-means clustering were in a good agreement in grouping the genotypes into five distinct clusters (Supplementary Figure S2, Figure  3B). Clustering did not follow cultivation classification as winter *vs* spring type, except that cluster 5 was largely dominated (82%) by winter triticale.

Further analysis using DAPC was conducted to capture more genetic variation. Result from DAPC showed that about 95% of the total genetic variation was explained by the first 200 PCs (Figure  3B inset, PCA eigenvalues). The first three axes of discriminant analysis (DA) captured most of the subpopulation structures of the total population. The first discriminant axis (DA1) separated cluster 5 far from the rest of the clusters (Figure  3B). The DAPC analysis helps identify SNPs that are directionally selected relative to the clusters identified. Markers that had the highest loadings on DA1 were located on groups 1, 2, 5, and 6 (Supplementary Figure S3). These markers had higher frequency in winter than in spring triticale.

### Variation for forage yield

There was high and erratic field heterogeneity that was corrected with spatial smoothing using 2D tensor product splines (Supplementary Figure S4). Spatial analysis showed about 24.5% higher efficiency in reducing random error compared with lattice design. There was significant variation between genotypes, environments, and the interactions between the two. Broad sense heritability was 42% and 47% for lattice and spatial analysis, respectively. Best linear unbiased estimates were used for estimating GEBV of individuals.

### Genomic selection applications

**Figure 4:**
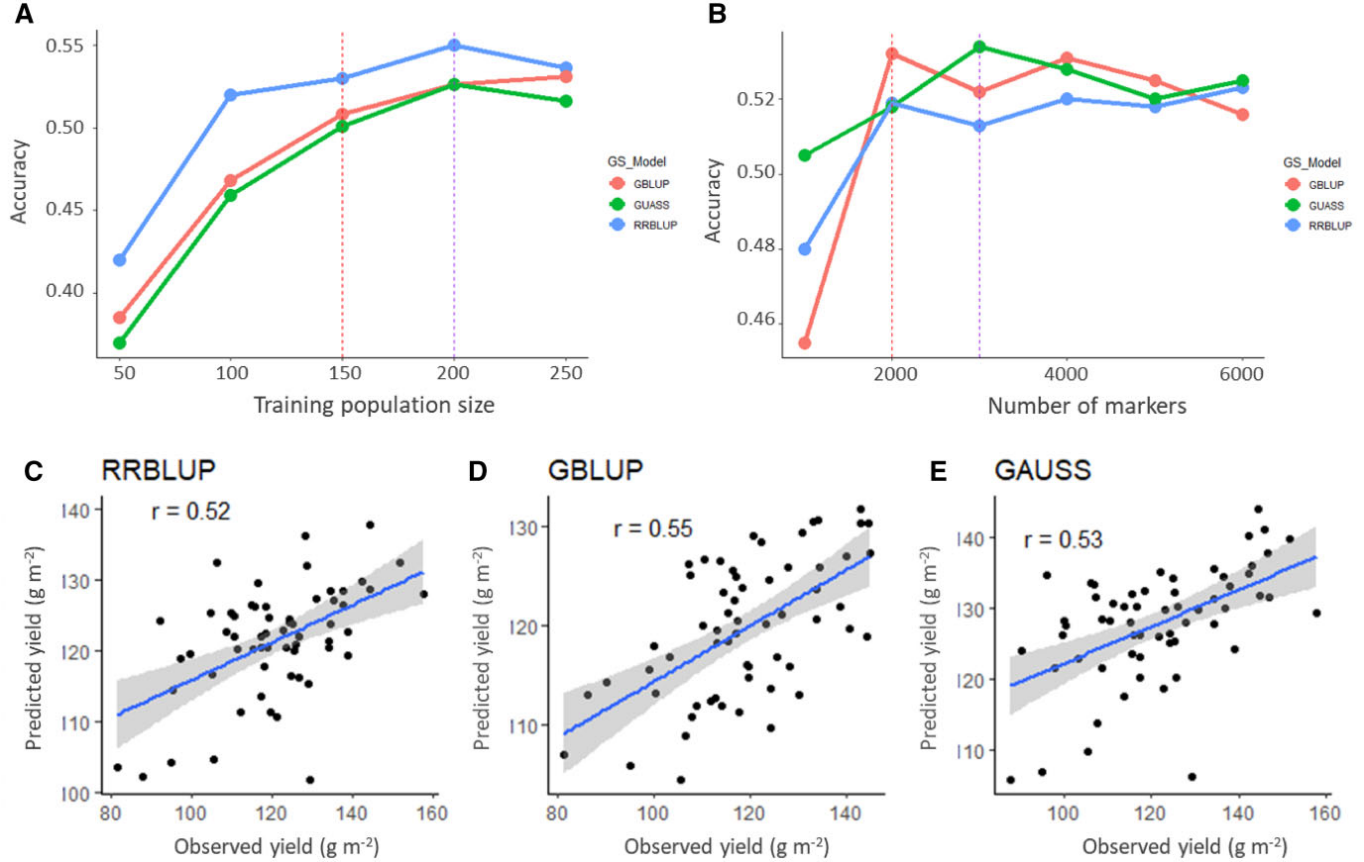
Genomic selection modeling of forage yield of the population. Genomic selection accuracy as a function of training population size when 7000 markers were used (A), and marker density when training population size was 200 (B). Scatterplots showing correlations between the observed (PEBV) and the predicted values (GEBV) of forage yield using the three models (C–E). The shaded area shows the 95% confidence interval of the correlation line (blue).

Individuals and markers were randomly assigned into training and validation populations. Prediction accuracy of forage yield (dry matter) increased with increasing training population size when a large number (7000) of markers were used (Figure  4A). The RRBLUP model showed relatively higher accuracy, but its trend of increase was fluctuating relative to the size of training population (Figure  4A). The other two models, GBLUP and GUASS, were similar in performance with minimum fluctuations when the size of training population was increased up to 200. However, gain in accuracy tended to plateau (or even decrease) for all three models when the training population size was beyond 200. As a result, the optimal marker density was determined using 200 training population size. Similar to training population size, increasing marker density improved GS accuracy (Figure  4B). However, increasing marker density beyond 2000 did not show any substantial increase in prediction accuracy (Figure  4B). There was a tendency of overfitting when more than 3000 markers were used while the training population size was fixed to 200. Average selection accuracy of all three models was about 0.52 for forage yield when the models were trained with 200 genotypes and 2000 markers. Figure  4, C–E show scatter plot of GEBV and PEBV by the three models. From the scattering pattern of points, GBLUP was the most precise and consistent for this analysis (Figure  4D).

## Discussion

### Genotyping-by-sequencing enables efficient marker discovery in triticale

This study showed that GBS is an effective marker development technology for triticale breeding and genetics research. GBS is especially suited for genetic improvement of orphan crops like triticale through combined marker discovery and genotyping of large populations, even in the absence of a reference genome (Poland *et al.* 2012a). Reference genomes of closely related species like wheat can be used to develop markers for triticale. In this study, 45% of high-quality triticale SNPs were mapped to hexaploid wheat genome. Interestingly, a significant fraction of the SNPs was mapped to the D genome of hexaploid wheat. This result indicated that some of the triticale accessions in this study were substituted triticale lines, which were derived from crosses between hexaploid wheat and triticale (Gustafson *et al.* 1989; Hao *et al.* 2013). The percentage of SNPs that were mapped to the R genome was much higher than that of mapped to A and B genomes combined. This should be attributed to the out-crossing rye progenitor and recently released rye reference sequence (Rabanus-Wallace *et al.* 2021). Previously, Kuleung *et al.* (2004) reported that 57% of wheat and 39% of rye simple sequence repeat (SSR) markers were transferable to triticale. Transferability of markers seemed also to depend on the types of marker technologies used and diversity of the population studied. Badea *et al.* (2011) reported that 24% of diversity array technology (DArT) markers originating from rye, and only 9% from wheat genomes were polymorphic on triticale. Allopolyploidization of the two parental genomes was reported to cause sequence modifications or losses of 10–30% in wheat and up to 50% in rye genomes (Boyko *et al.* 1984; Ma *et al.* 2004; Ma and Gustafson 2008), which might partly explain these discrepancies. Moderate transferability of markers (45% to wheat and 58% to rye) was observed in the present study, even though the initial marker density was very high compared with previous studies.

### Linkage disequilibrium decayed in a relatively short genomic distance

LD is a population specific parameter commonly estimated using a squared value of the correlation coefficient (*r*^2^ statistics) of the allelic states of two given polymorphic loci (Hill and Weir 1988). The extent of LD in a population determines the number of markers, and experimental design to be used for a successful genome-wide association analysis (Flint-Garcia *et al.* 2003). LD decayed very quickly to *r*^2^ < 0.1 over 10 kb, indicating the absence of long LD blocks shared in this population. The relatively quick LD decay in this population might be due to the slightly open pollinating nature of triticale which can be up to 10% (Oettler 2005). The three genomes showed comparable LD decay distances and percentage of markers that were in LD. Introduction of chromosomes from different ancestries often results in LD that breaks down rapidly with random mating (Pritchard and Rosenberg 1999). The decline of LD with distance is generally affected by nonrandom mating, selection, mutation, migration or admixture, genetic drift, and the effective population size (Flint-Garcia *et al.* 2003). Short LD blocks in this population bear a potential for high resolution GWAS because long LD blocks increase frequency of false positive associations (Balding 2006; Otyama *et al.* 2019).

### Large genetic diversity exists in the triticale panel

Population structure was analyzed using three different methodologies including hierarchical clustering, *k*-means clustering and PCA. The three methods grouped the population into five clusters with similar subpopulation memberships. The first three PCs of the PCA only explained 19% of variation. Information in the first three PCs showed structuring patterns emerging, but it also did not use the larger proportion of genomic information in remaining PCs (Figure  2). Therefore, PCs were further analyzed using DAPC to extract more information from nearly all of the PCs (Jombart *et al.* 2010; Ayalew *et al.* 2020). In addition to the larger amount of information used in DAPC, it enables to interpret loading of individual markers along discriminant axes (Supplementary Figure S3). *K*-means clustering provides statistical validation to decide optimum cluster number during hierarchical clustering, which is mostly subjective otherwise. Clustering in this population did not follow the winter—spring cultivation classification except for cluster 5 which was largely dominated by winter types (82%). *K*-means clustering handles large dataset better compared with hierarchical clustering (Jombart *et al.* 2010).

### Genomic selection shows a good potential in selecting for forage yield in triticale

GS accuracy increased with increasing training population size. This was in agreement with previous studies (Norman *et al.* 2018; Maulana *et al.* 2021). However, the rate of increase slowed down beyond 150 lines in the training population (Figure  4A). In addition to training population size, heritability of the trait, extent of LD, physical distance, and genetic relationship matrix also affect GS accuracy depending on the underlying assumptions of models used (Solberg *et al.* 2008; Zhong *et al.* 2009; Habier *et al.* 2010). LD decayed in a relatively short physical distance, which negatively affects the prediction accuracy. This is usually the case when individuals are distantly related in a diverse population.

The moderate level of GS efficiency in this study is encouraging to incorporate GS in forage triticale breeding programs. A reasonable level of accuracy can be achieved by using training population size of as low as 100 individuals but the most optimal number of training size is 200 in terms of gain in accuracy relative to size of training population (Figure  4A). The RRBLUP model performed very well when a large number (7000) of markers were used (Figure  4A), however, its performance was not ideal compared to GBLUP when the number of markers were reduced to 2000 (Figure  4B). Prediction accuracy did not increase beyond 2000 markers for RRBLUP and GBLUP models, and 3000 markers for GUASS when training population size was fixed to 200 (Figure  4B). Therefore, 200 training individuals and 2000 markers gave optimal combination for predicating forage yield in this population. As most genotypes in the population are parents of our breeding program, this study provides immediate guidelines for structuring our GS strategies in forage triticale breeding.

Reducing or controlling field heterogeneity is one of the long standing experimental design principles in agricultural research (Gilmour *et al.* 1997; Rodríguez-Álvarez *et al.* 2018). In addition to the number and quality of markers, the quality of the phenotype data used in GS models is equally important for successfully applying GS in breeding. We compared the efficacies of lattice design and spatial analyses techniques for their ability to reduce residual error. The use of spatial analysis in this study showed a 24.5% reduction in residual error, and a 12% increase in heritability estimate. Heritability is one of the main factors that affect predict ability of GS.

## Conclusion

Even though triticale is a new species with a very short history of development, we found large genetic diversity in this population. LD declined quickly partly because triticale has some degree of out crossing and partly because of the introduction of chromosomes from different ancestries that breaks down rapidly with random mating and genomic mixing. This makes triticale amenable for high-resolution GWAS analysis. Average GS accuracy for forage yield was 0.52 with 200 training individuals and 2000 SNPs, which is encouraging for such a quantitative trait. The result indicates that GS can facilitate triticale breeding for forage yield improvement. In conclusion, this panel has large genetic diversity that can be exploited though genome mapping and be used for selective breeding.

## Data availability

All Supplementary materials and relevant data are available at figshare at https://doi.org/10.25387/g3.14233568. Raw sequence reads were deposited at NCBI’s Sequence Read Archive (SRA) under project PRJNA715663 and will be accessible upon release at http://www.ncbi.nlm.nih.gov/bioproject/715663.
